# The Akt/FoxO/p27^Kip1^ axis contributes to the anti‐proliferation of pentoxifylline in hypertrophic scars

**DOI:** 10.1111/jcmm.14498

**Published:** 2019-07-03

**Authors:** Fangfang Yang, Erfei Chen, Yunshu Yang, Fu Han, Shichao Han, Gaofeng Wu, Min Zhang, Jian Zhang, Juntao Han, Linlin Su, Dahai Hu

**Affiliations:** ^1^ Department of Burns and Cutaneous Surgery Xijing Hospital, Fourth Military Medical University Xi'an, Shaanxi Province China; ^2^ Institute of Preventive Genomic Medicine, School of Life Sciences Northwest University Xi’an China

**Keywords:** anti‐proliferation, hypertrophic scars, p27^Kip1^, pentoxifylline

## Abstract

Hypertrophic scars (HS) are characterized by the excessive production and deposition of extracellular matrix (ECM) proteins. Pentoxifylline (PTX), a xanthine derived antioxidant, inhibits the proliferation, inflammation and ECM accumulation of HS. In this study, we aimed to explore the effect of PTX on HS and further clarify the mechanism of PTX‐induced anti‐proliferation. We found that PTX could significantly attenuate proliferation of HS fibroblasts and fibrosis in an animal HS model. PTX inhibited the proliferation of HSFs in a dose‐ and time‐dependent manner, and this growth inhibition was mainly mediated by cell cycle arrest. Transcriptome sequencing showed that PTX affects HS formation through the PI3K/Akt/FoxO1 signalling pathway to activate p27^Kip1^. PTX down‐regulated p‐Akt and up‐regulated p‐FoxO1 in TGF‐β1 stimulated fibroblasts at the protein level, and simultaneously, the expression of p27^Kip1^ was activated. In a mouse model of HS, PTX treatment resulted in the ordering of collagen fibres. The results revealed that PTX regulates TGFβ1‐induced fibroblast activation and inhibits excessive scar formation. Therefore, PTX is a promising agent for the treatment of HS formation.

## 1 INTRODUCTION

Cutaneous scarring is a pathognomonic feature that emerges following burns to the skin and characteristically underlies post‐burn physical and psychosocial morbidity. The most common cicatrix formed following a burn is the hypertrophic scar (HS), the prevalence of which has been reported to be as high as 70%. Patients with these massive burns have extensive scarring and contractures, itch and pain.^1^ In severely‐burned patients, HS are the most common type of scar. During the wound healing process, fibroblasts are a vital type of effector cells that upon activation induces synthesis of the extracellular matrix (ECM) and inflammatory cytokines. However, the mechanism of HS formation is far from clear. Recently, it was reported that the abnormal expression of several cytokines is associated with hypertrophic scar formation. Above all, TGF‐β1 is the cytokine associated with fibrotic disease and hypertrophic scarring. Involving multiple cellular processes, TGF‐β1 regulates tissue homeostasis, including cell proliferation, migration, apoptosis and ECM remodelling.^2^, ^3^

Several treatments are used to reverse scar formation and emulate normal wound healing and remodelling, including transdermal injections, biomaterial‐based approaches and non‐pharmacological approaches.^4^ However, these methods are inadequate in reducing or preventing scar formation. Pentoxifylline (PTX) is a xanthine derived antioxidant that takes potent inhibitory action against cell proliferation, inflammation and ECM accumulation.^5^ Research has increasingly suggested that PTX markedly reduces proteinuria in patients with membranous nephropathy or diabetes.^6^ Moreover, PTX has been shown to enhance the antitumour effect and to sensitize tumour cells to radiotherapy.^7^, ^8^ Previous research has shown that PTX has a direct effect on inhibiting the generation of burn scar fibroblasts. However, the mechanism by which PTX affects HS formation is not fully understood. In this study, we investigated the mechanism by which PTX inhibited the synthesis of HSFs and TGF‐β1‐induced normal dermal fibroblasts (NFs). Our results indicate that PTX inhibits the expression of collagen Ⅰ (Col1), collagen Ⅲ (Col3) and α‐SMA in HS fibroblasts. RNA sequencing showed that the inhibitory role of PTX on HSFs is mediated by the activation of p27^Kip1^ through Akt/FoxO pathway.

## 2 MATERIALS AND METHODS

### 2.1 Ethics statement

All experimental procedures, including the use of human and animal samples, were conducted under a protocol (No: XJYYLL‐2013109) reviewed and approved by the Institutional Ethical Committee of the Fourth Military Medical University.

### 2.2 Cell culture and treatment

Normal skin and hypertrophic scar tissue were taken from three patients who had not been treated for burns before surgery. The patients’ ages ranged from 18 to 44 years. Written consent was obtained from patients or their legal guardians. The samples were pruned to remove excess adipose tissue and rinsed with phosphate buffer (PBS) three times, minced into pieces and then incubated in Dulbecco's modified Eagle's medium (DMEM; Gibco, USA) containing 0.1% collagenase type I (Sigma‐Aldrich, USA) at 37°C for 3 hours. The isolated cells were cultured in DMEM containing 10% foetal calf serum (BI), 1% penicillin and 1% streptomycin, at 37°C in atmosphere of 5% CO_2_. The cell strains were maintained and analysed from passage 3 to passage 6. Fibroblasts were incubated to reach 70%‐80% confluence and then incubated in serum‐depleted medium for another 12 hours in preparation for treatment.

Several six‐well culture plates of HS‐derived fibroblasts were divided into five groups (n = 3) with different concentrations of PTX, 0 mg/mL (control), 0.25 mg/mL, 0.5 mg/mL, 1 mg/mL and 2 mg/mL, and cultured for 24 hours and 48 hours. Recombinant human TGF‐β1 was purchased from PeproTech (London, UK) and dissolved in 10 mmol/L citric acid (pH 3.0), yielding a final stock concentration of 10 ng/mL.

### 2.3 Histology staining

Hypertrophic scar and normal skin samples were fixed with 4% paraformaldehyde. 4‐μm‐thick sections were used for haematoxylin‐eosin (HE) and Masson staining.

### 2.4 RNA extraction and quantitative real‐time PCR

Total RNA was extracted from human scar‐derived fibroblasts using TRIzol reagent (Invitrogen, USA). Isolated RNA was reverse transcribed to cDNA (Takara, Japan). Quantitative PCR was performed using Bestar SybGreen qPCR MasterMix (DBI Bioscience, German) by the CFX96^TM^ Real‐Time PCR detection system. A total volume of 10 μL was subjected to the PCR regimen: 95°C, 2 minutes for initial denaturation, 40 cycles of 95°C for 10 seconds and 60°C for 30 seconds. Quantification was adjusted using the house‐keeping genes GAPDH, and relative expression was calculated according to the formulas 2^‐ΔΔCt^. Primers are shown in Table S1.

### 2.5 Western blot analysis

To obtain total protein, cells were lysed in RIPA buffer with protease inhibitor added. Equal amounts of protein (20 or 30 μg) were separated on a 10% SDS‐PAGE gel and then electroblotted onto PVDF membranes. After blocking with 5% non‐fat‐dried milk in Tris‐buffered saline with 0.1% Tween 20 (TBST) buffer for 1 hours at room temperature, the membranes were incubated overnight at 4°C with primary antibodies. The membranes were washed and incubated with corresponding IgG‐HRP secondary antibody (Jackson ImmunoResearch, USA). The bands were visualized with the AlphaImager HP system. The antibodies used in this study were as follows: anti‐Col1 (Abcam, ab96723, 1:2000), anti‐Col3 (Abcam, ab184993, 1:1000), anti‐α‐SMA (Proteintech, 14395‐1‐AP, 1:1000), anti‐Akt (CST, #4691, 1:1000), anti‐pAkt (CST, #4060, 1:1000), anti‐FoxO1 (CST, #14952, 1:1000), anti‐pFoxO1(Thr24)/FoxO3a (Thr32) (CST, #9464, 1:1000), anti‐p27^Kip1^ (CST, #3686, 1:1000) and anti‐GAPDH (Bioss, bs‐2188R, 1:4000).

### 2.6 Transcriptome sequencing and analysis

After PTX treatment with HSFs, total RNA was extracted using TRIzol reagent (Invitrogen, USA). RNA‐seq and bioinformatics data were analysed by Shanghai Novelbio Ltd. The DEseq algorithm was applied to filter the differentially expressed genes, after the analysis for significance, following criteria that fold changes >2 or <0.5. The resulting *P* values were adjusted through use of the Benjamini and Hochberg (BH) FDR algorithm. The significant enrichment pathway was found by a Fisher exact test. Gene co‐expression networks were built in accordance with the normalized signal intensity of genes with specific expression levels. The sequencing data acquired in this study have been submitted to the NCBI Database of GEO datasets (https://www.ncbi.nlm.nih.gov/geo) under accession number GSE 122891.

### 2.7 CCK‐8 assay

Cell Counting Kit‐8 (Dojindo Laboratories, Japan) was performed according to a previously reported protocol.^9^, ^10^ Four replicate sets of treated cells were inoculated at a density of 3,000 cells/well into a 96‐well plate. To measure the proliferative activity, 10 μL CCK‐8 solution and 90 μL of complete medium were added to each well. After incubation at 37°C for 1 hours, the number of viable cells was measured at a wavelength of 450 nm.

### 2.8 Flow cytometry analysis

The cell cycle for each sample was detected using PI/RNase staining buffer (BD Biosciences, USA) according to the manufacturer's instructions. HS‐derived fibroblasts were treated with DMEM (control) or 2 mg/mL PTX for 24 hours. Cells were digested with 0.25% trypsin and then washed twice with cold PBS. After cells were fixed and permeabilized with 70% ethanol fixing and permeabilizing cells, 0.5 mL/test of the fixing mixture with cells was incubated for 15 minutes at room temperature before analysis.^11^ For the apoptosis assay, the cells were tested with FITC Annexin V apoptosis detection kit I (BD Biosciences, USA) as previously described, resuspended in binding buffer and incubated with Annexin V and PI for 15 minutes at room temperature in the dark.

### 2.9 Animal model

Eight‐week‐old Balb/c mice (n = 18) were housed under standard conditions. Mice were randomly divided into three groups and with 6 mice/group: control group (PBS injected), PTX injected (25 mg/kg) and PTX injected (50 mg/kg). According to the protocol provided by Alexander M. Cameron et al and Tatsuo Maeda eta al,^12^, ^13^, ^14^ we used the bleomycin‐induced method to establish the in vivo hypertrophic scar model. One hundred microliters of bleomycin (500 μg/mL, Selleck) or phosphate‐buffered saline (PBS) (control) was subcutaneously injected into the back skin of mice (n = 18) for 6 continuous days per week for a total of 8 weeks.

### 2.10 Statistical analysis

The results were presented as the means ± SEM. GraphPad Prism (GraphPad Software, Inc) was used for unpaired *t* tests or paired *t* tests. A *P* value <0.05 was considered statistically significant.

## 3 RESULTS

### 3.1 PTX down‐regulates mRNA and protein levels of several fibrosis‐related molecules in a dose‐ and time‐dependent manner

**Figure 1:**
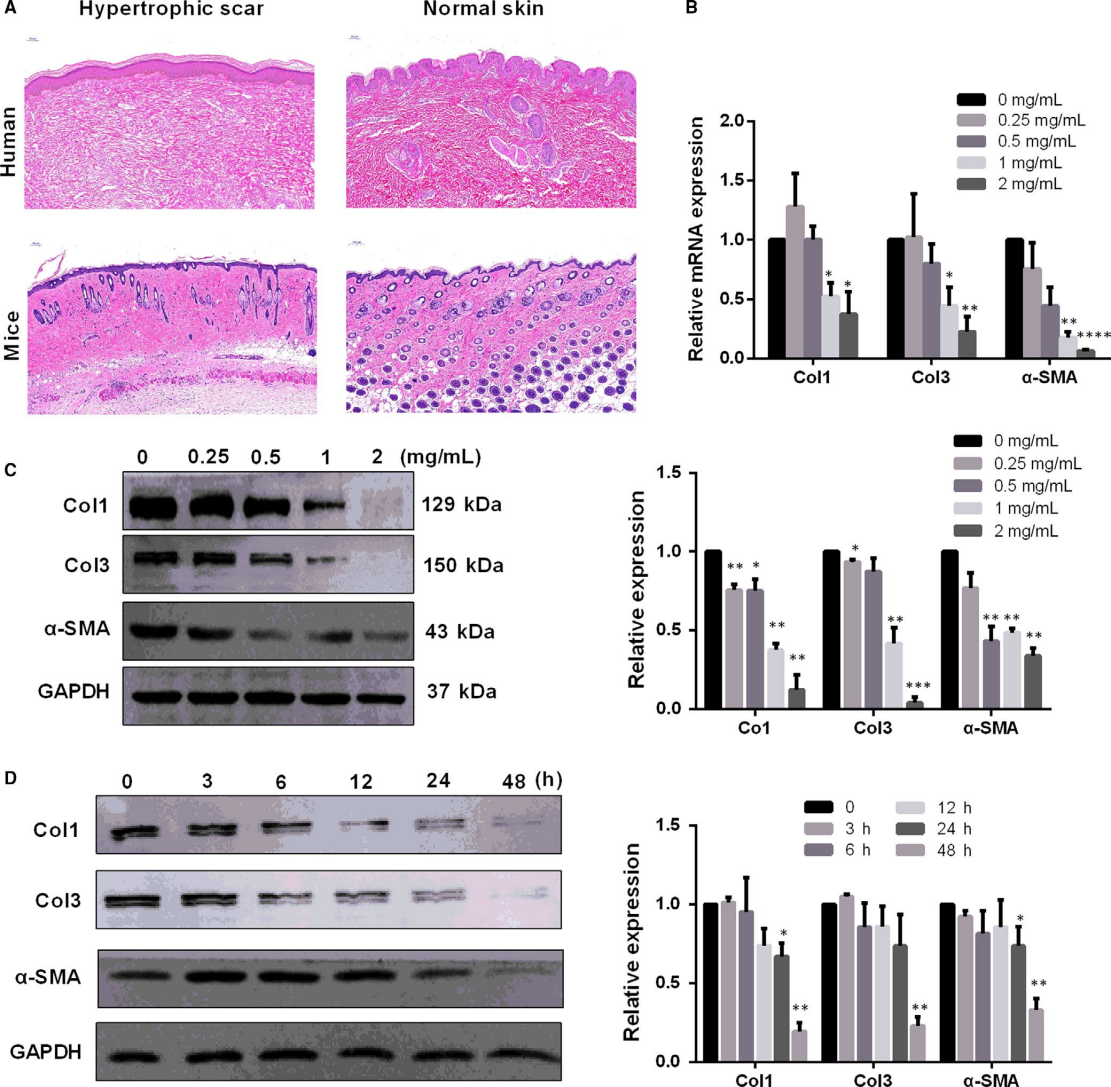
Effects of PTX on mRNA and protein levels of ColI, ColIII and a‐SMA in HS fibroblasts. (A) Bleomycin was delivered subcutaneously, and the histology of fibrotic lesions was similar to that of human hypertrophic scars (dermal thickening, hypertrophic epidermis, increased cellularity and reduction in the number of dermal appendages); scale bar = 100 μm. (B) Histogram summarizes qRT‐PCR data on the mRNA levels of Col1, Col3 and a‐SMA after PTX treatment at different concentrations: 0, 0.25, 0.5, 1 and 2 mg/mL. GAPDH was used as an internal control. (C) Western blot analysis of Col1, Col3 and a‐SMA protein expression after PTX treatment. GAPDH served as an equal protein loading control. (D) Western blot analysis of Col1, Col3 and a‐SMA protein expression after PTX treatment at different time points. The results were based on four independent experiments using cells from four different HS patients. Each data point was normalized according to its corresponding GAPDH level and then normalized according to the value of the control (0 mg/mL PTX), which was arbitrarily set as 1. Each bar represents the mean ± SEM of n = 4. *P < 0.05; **P < 0.01, compared to control

The fibrotic lesions were histologically similar to those observed in human hypertrophic scars treated with bleomycin (increased cell and reduced the number of dermal appendages, dermal thickening and hyperplastic epidermis, Figure 1A). As has been extensively reported, the expression of fibrosis‐related molecules Col1, Col3 and α‐SMA in HS is up‐regulated.^15^, ^16^ To investigate the suppressive effect of PTX on HS formation, we measured the expression of Col1, Col3 and α‐SMA in HS‐derived fibroblasts that were isolated and treated with PTX at different concentrations of 0, 0.25, 0.5, 1 and 2 mg/mL. QRT‐PCR revealed that all three marker molecules were robustly reduced in HSFs following treatment with increasing concentrations of PTX (Figure 1B). PTX remarkably down‐regulated Col1, Col3 and α‐SMA mRNA levels at concentrations of 1 mg/mL and 2 mg/mL (*P < 0.05, **P < 0.01, ****P < 0.0001). At the protein level, the optimum concentration was 2 mg/mL. PTX almost completely inhibited the expression of Col1 and Col3. Therefore, the concentration of PTX at 2 mg/mL was chosen and used in the subsequent experiments. Accordingly, expression response to PTX was tested for time‐dependent effectiveness: treatment with PTX at 2 mg/mL after 3, 6, 12, 24 and 48 hours. Western blotting showed that all three marker molecules were significantly inhibited at 48 hours (**P < 0.01, ***P < 0.001, Figure 1D).

### 3.2 PTX inhibited the proliferation of HS fibroblasts via cell cycle arrest

**Figure 2:**
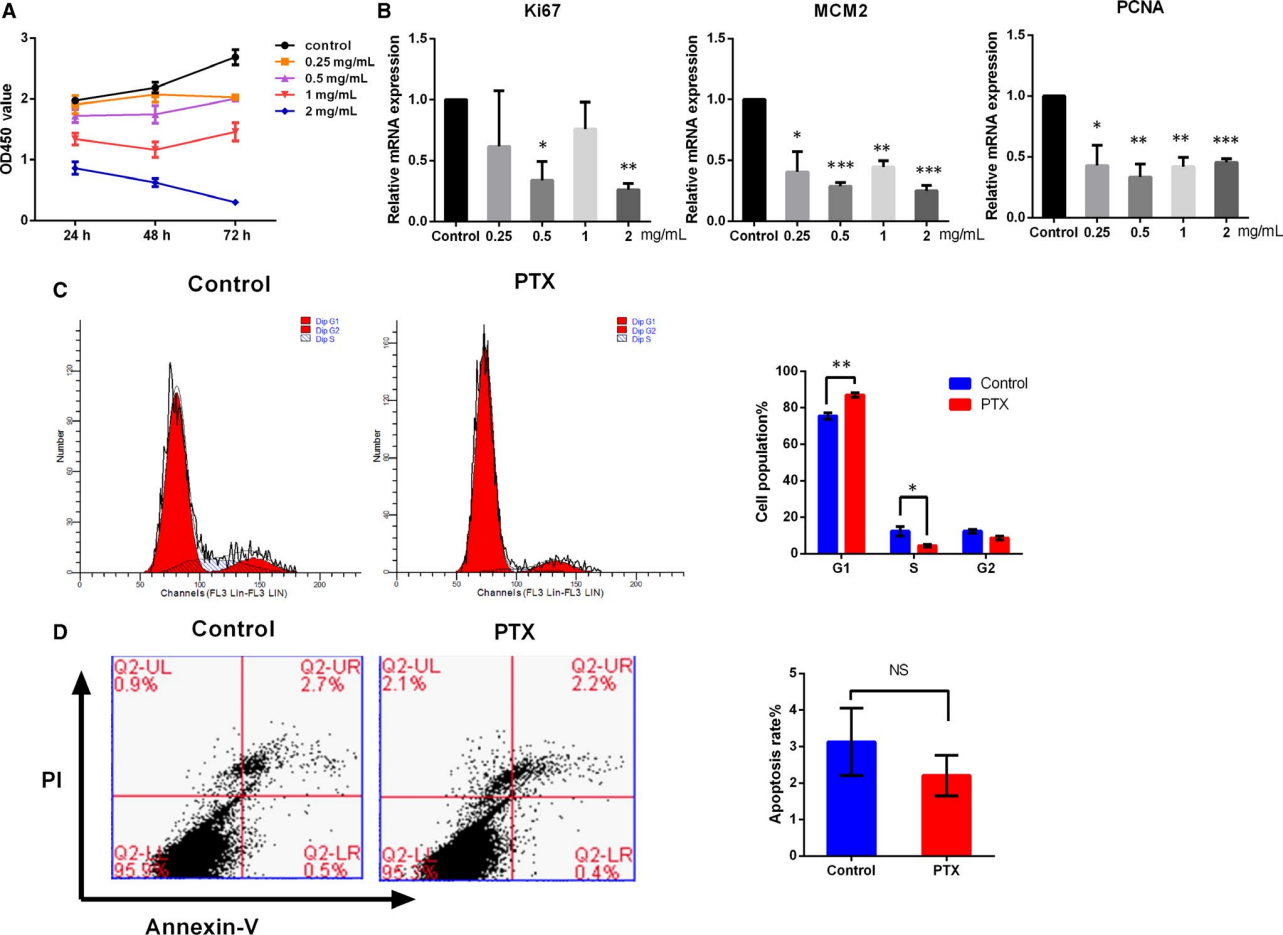
PTX inhibited the proliferation of HS fibroblasts. (A) CCK8 assays were used to determine cell viability. (B) QPCR analysis of proliferation markers Ki67, MCM2 and PCNA in cells treated with different concentrations of PTX. (C‐D) Cell cycle distributions and apoptosis were detected by flow cytometry. Representative data from triplicate experiments are shown. All data are shown as the mean ± SEM, *P < 0.05, **P < 0.01, ***P < 0.001

In our study, CCK8 assays showed that the PTX significantly inhibited HS fibroblasts proliferation, especially at a concentration of 2 mg/mL (Figure 2A). In addition, the expression levels of biomarkers Ki67, MCM2 and PCNA were significantly decreased 24 hours after PTX treatment (Figure 2B). To further illustrate the growth inhibitory effect of PTX, cell cycle and apoptosis were measured and the results revealed that PTX promoted G1 phase arrest (*P < 0.05, **P < 0.01, Figure 2C), whereas apoptosis assays showed no difference between the control and PTX groups (Figure 2D).

### 3.3 A novel downstream network of PTX characterized by RNA sequencing

**Figure 3:**
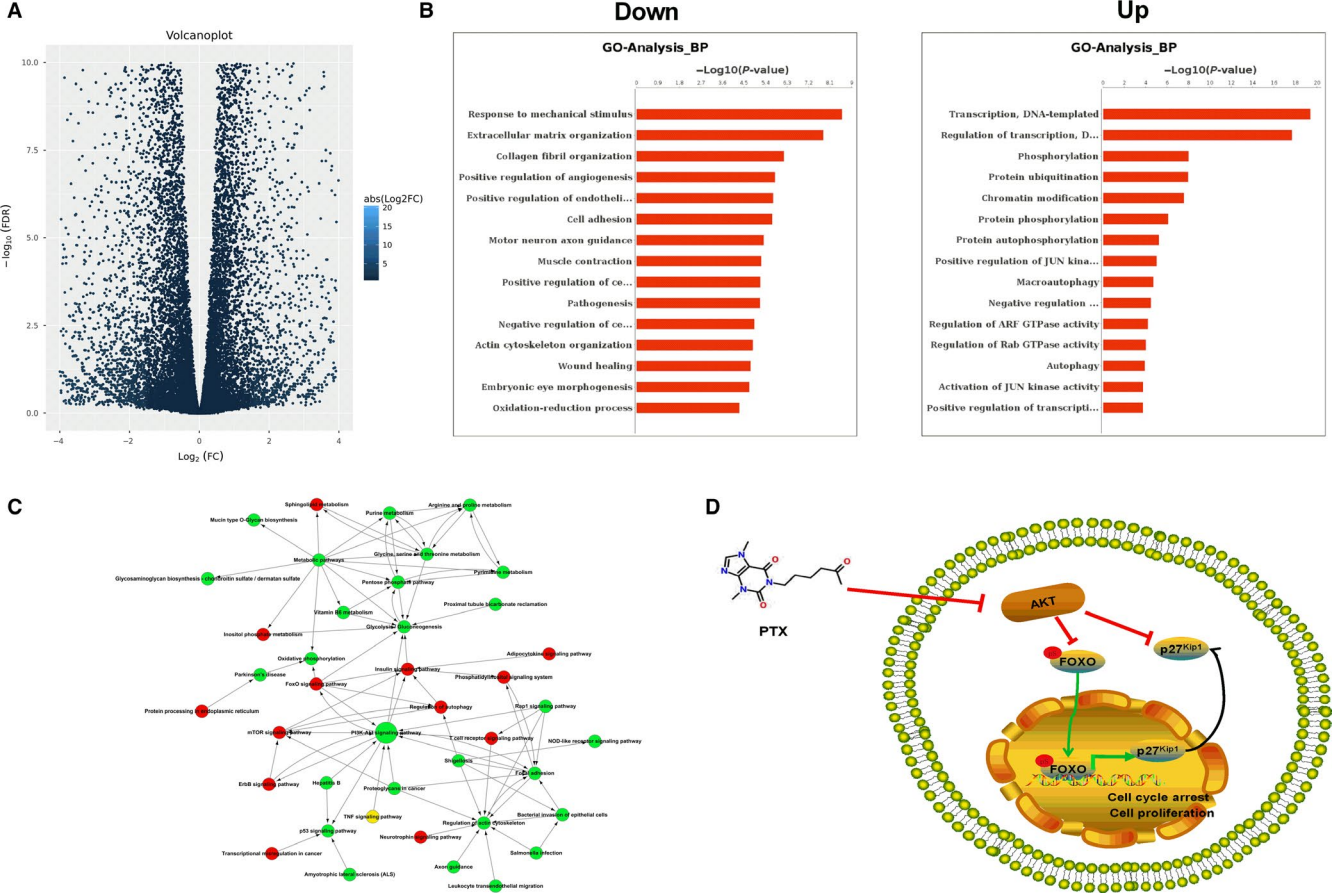
Transcriptome profile identifies differentially expressed genes and pathways induced by PTX. (A) The volcano plot of differentially expressed genes analysed by RNA‐seq (fold change >2 or <0.5). (B) GO analysis of differentially expressed genes. Left: down‐regulated genes. Right: up‐regulated genes. (C) Pathway network analysis of differentially expressed genes. The Fisher exact test was used to find the significant enrichment GO term or pathway. (D) Schematic model of PTX‐induced inhibition of Akt and activation of the p27^Kip1^ signalling pathway

In this study, we aimed to characterize the downstream gene and pathway changes induced by PTX. RNA sequencing data revealed 2625 up‐regulated and 2683 down‐regulated genes, with > twofold change and FDR <0.05 compared to the control group (Figure 3A). GO analysis showed that the down‐regulated genes were mainly correlated with response to mechanical stimulus (eg IL6 and CCL2), collagen fibril organization (eg Col3A1, Col1A1 and Col1A2), positive regulation of angiogenesis (eg FGF1, FGF2 and IL1B), and wound healing (eg IL6 and TGFB2), whereas up‐regulated genes were mainly correlated with phosphorylation (Figure 3B). Pathway analysis showed these genes were enriched in the focal adhesion, TNF, PI3K‐Akt, FoxO and p53 signalling pathways (Figure 3C). We further investigated gene co‐expression networks to reveal the interactions among genes and to select the key genes regulated by PTX (Figure S1). We found p27^Kip1^ (CDKN1B), a cyclin‐dependent kinase inhibitor, is one of the core regulatory genes in PTX‐treated cells. Together with the results from the pathway network analysis, we hypothesized that upregulation of p27^Kip1^ activated by FoxO1 could be a novel mechanism regulated by PTX. Akt2 was confirmed to be down‐regulated in PTX‐treated cells (Log_2_FC = −1.68, FDR = 8.55E‐64), thus further alleviating the inhibitory effect on FoxO (Figure 3D).

### 3.4 PTX down‐regulates the PI3K/Akt/FoxO pathway in TGF‐β1‐mediated fibrosis

**Figure 4:**
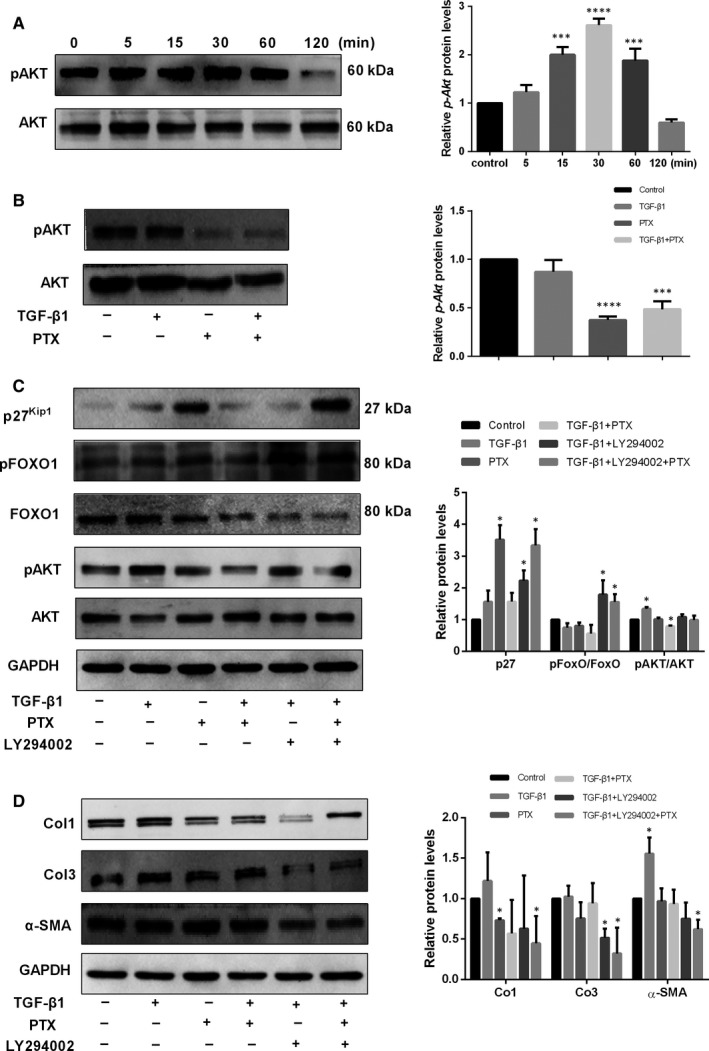
PTX down‐regulated the expression of p‐Akt in TGF‐β1‐stimulated normal fibroblasts. Fibroblasts were divided into six groups and stimulated with TGF‐β1. (A) TGF‐β1 significantly increased Akt phosphorylation 30 min after stimulation. Next, fibroblasts were divided into four groups and stimulated with TGF‐β1 (10 ng/ml), PTX (2 mg/mL), both or neither. (B and C) PTX significantly reduced the phosphorylation of Akt that was induced by TGF‐β1. Then, fibroblasts were divided into six groups and treated with TGF‐β1 (10 ng/mL), PTX (2 mg/mL), TGF‐β1 (10 ng/mL) + PTX (2 mg/mL), TGF‐β1 (10 ng/mL) + LY294002 (50 μmol/L), TGF‐β1 (10 ng/mL) + PTX (2 mg/mL) + LY294002 (50 μmol/L) or dimethyl sulfoxide (DMSO, control). (D) LY294002 or PTX significantly inhibited the expression of Col1, Col3 and α‐SMA that was induced by TGF‐β1 (*P* < 0.05) (n = 4)

PI3K/Akt is constitutively activated in the majority of hypertrophic scars.^17^ According to the results of sequencing, we found that inhibition of hypertrophic scar growth by PTX was because of inhibition of the PI3K/Akt pathway and activation of p27^Kip1^. To confirm this finding, constitutive levels of Akt and FoxO1 were examined in NFs stimulated by TGF‐β1, LY294002 and PTX treatment. We examined whether TGF‐β1 stimulation could increase the phosphorylation of p‐Akt in normal skin‐derived fibroblasts. As shown in Figure 4A, the expression of p‐Akt peaked 30 minutes after stimulation with TGF‐β1. Next, we investigated the downstream molecule FoxO1 and its target, p27^Kip1^, which play vital roles in fibrosis. Our results showed that PTX suppressed the upregulation of p‐Akt, Col1, Col3 and α‐SMA induced by TGF‐β1. Furthermore, the levels of FoxO1‐regulated p27^Kip1^ were substantially increased in PTX‐treated cells compared to control cells (Figure 4C), indicating that the PI3K/Akt/FoxO pathway plays a critical role in TGF‐β1‐mediated skin fibrosis. During this process, the expression of Col1, Col3 and α‐SMA was reduced. The effect of LY294002, an inhibitor of the PI3K/Akt pathway, was similar to that of PTX (Figure 4D).

### 3.5 PTX attenuates excessive scarring in the mice HS model

**Figure 5:**
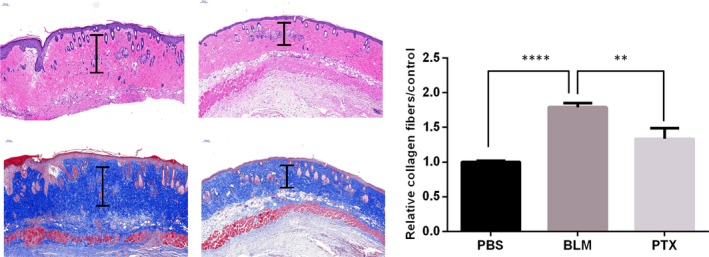
PTX attenuates excessive scarring in the mouse HS model. HE staining showed that the hypertrophic scar features such as dermal thickening, hyperplastic epidermis, increased cellularity and reduction in the number of dermal appendages were significantly reversed after PTX treatment; scale bar = 100 μm. Collagen deposition was attenuated in the PTX‐treated mice scars as shown by Masson staining; scale bar = 100 μm. Chart shows the dermal thickness induced by bleomycin and after PTX treatment, **P < 0.01, ***P < 0.001

To evaluate the regulation of PTX on hypertrophic scar formation, we used HE staining and found that HS features such as thickening of the dermis, hyperplastic epidermis, increased cellularity and reduction in the number of dermal appendages were significantly reversed after PTX injection (50 mg/kg).^18^ Masson staining showed that scar collagen deposition in mice after local injection of PTX was also attenuated (*P* < 0.001, Figure 5), whereas local injection of PTX (25 mg/kg) did not thicken the dermis after scar formation. These results suggested that PTX might attenuate hypertrophic scarring in vivo.

## 4 DISCUSSION

In this study, we found that PTX inhibited the progression of HSFs. We discovered that PTX down‐regulated Col1, Col3 and α‐SMA. The anti‐HS activities of PTX were associated with the inhibition of intracellular PI3K/Akt/FoxO signalling and the subsequent upregulation of p27^Kip1^. Consistent with the effects on HSFs in vitro, PTX potently reduced collagen deposition, α‐SMA expression, and promoted HS healing in vivo. These results collectively suggest that PTX attenuates the phenotype of HSFs and is a promising therapeutic agent for treating HS.

Pentoxifylline (PTX) is a methylxanthine derivative and has been extensively studied in a variety of disease models. It induces vascular dilation and increases erythrocyte flexibility resulting in enhanced blood flow.^19^ PTX indirectly inhibits angiogenesis in mouse pro‐epicardial explant cultures but has no significant effect on the C166 endothelial cell line.^20^ It also has anti‐tumour necrosis factor α activity and is believed to reduce the cytokine cascade. PTX was shown to significantly affect the wound healing process in *streptozotocin*‐induced diabetes in rats.^21^ Similarly, it accelerates the wound healing process by modulating the gene expression of MMP‐1, MMP‐3 and TIMP‐1 in Normoglycemic rats. Furthermore, PTX is an adjuvant treatment for perioral post‐burn hypertrophic scars that may exert an anti‐fibrogenic effect by reducing cell proliferation, thereby decreasing the synthesis of extracellular matrix components. It was effective in improving HS elasticity, but the mechanism of PTX is not known.^21^, ^22^ PTX reduced the proliferation and contraction of burn scar fibroblasts in a dose dependent manner in (monolayer and fibroblast populated collagen lattice) FPCL models.^23^ PTX selectively inhibited Col3 synthesis in the HSF group, but inhibition was more pronounced for type I collagen synthesis in the NSF group.^24^ HS is mainly characterized by collagen deposition that is similar to the bleomycin‐induced HS mouse model. We observed that dermal thickening in vivo was significantly alleviated following PTX treatment. This result might be because of reduced collagen deposition, as shown by Masson staining.

Recently, Smad7 was reported to reverse TGF‐β1 transgene‐induced inflammation, fibrosis and the subsequently formed epidermal hyperplasia.^2^ TGF‐β1 is a proproliferative factor in the TGF‐β1‐Stat3‐FoxO1 axis in vascular smooth muscle cells.^25^ TGF‐β1 resulted in the acquisition of a myofibroblast‐like morphology and contractile phenotype and downregulation of endothelial markers in parallel with the induction of mesenchymal markers.^26^ Multiple signalling pathways have been identified as regulators of fibrosis, and PI3K/Akt signalling has been characterized in HS development. Numerous studies and reports have documented that high concentrations of TGF‐β within the wound area may be responsible for signalling through the PI3K/Akt pathway. In this study, pathway analysis suggested the potential significance of PI3K/Akt/FoxO signalling in PTX‐treated HSFs. Consistent with this result, we validated that PI3K/Akt/FoxO signalling significantly contributes to HS‐related fibrosis development in vitro. We also showed that in NSF treatments that included TGF‐β1, the PI3K/AKT pathway inhibitor LY294002 and PTX further decreased AKT phosphorylation, Col1, Col3 and α‐SMA expression and further increased pFoxO1 and p27 (Figure 4), suggesting that PI3K/Akt/FoxO signalling contributes to HS proliferation. Moreover, PTX induced inhibition of Akt/FoxO activation in HSFs.

p27^Kip1^ (CDKN1B) is a member of the Cip/Kip (CDK‐interacting protein/kinase inhibitor protein) family, which also includes p21 (CDKN1A) and p57 (CDKN1C).^27^ Initially discovered as a nuclear‐localized cell cycle inhibitor,^28^ p27^Kip1^ binds and inactivates CDKs and thus induces cell cycle arrest.^29^ The expression of p27 can be regulated by Akt directly or indirectly. PI3K activates Akt through phosphorylation, and active Akt further suppresses the expression of p27. In addition, activation of Akt can also inhibit the transcriptional activation of FoxO1 (one of the forkhead transcription factor class O (FoxO) isoforms) via phosphorylation‐dependent nuclear exclusion. As a direct target gene of FoxO1, the level of p27 changes, which affects cellular proliferation, apoptosis and differentiation.^30^

HS formation is characterized by the persistent activation of fibroblasts and the excessive production of extracellular matrix component collagen. Our study demonstrated that immunomodulatory PTX reduces HSF by targeting cell cycle progression, suppresses HSF activation by downregulating α‐SMA and collagen expression, and effectively treats HS in vivo. In summary, this study shows that PTX is a promising therapeutic agent for the treatment of HS.

## CONFLICT OF INTEREST

The authors state no conflicts of interest.
