# Supplementary material for: The Akt/FoxO/p27Kip1 axis contributes to the anti‐proliferation of pentoxifylline in hypertrophic scars
Source: J Cell Mol Med. 2019 Jul 3;23(9):6164–72. doi: 10.1111/jcmm.14498 (PMC6714140; doi:10.1111/jcmm.14498)
Supplement: Supplementary file 1 [file JCMM-23-6164-s001.docx]

**Supporting information**

Table S1

| Gene | Sequence |
| --- | --- |
| Col1（NM_000089.3） | F: GAGGGCAACAGCAGGTTCACTTA  R：TCAGCACCACCGATGTCCA |
| Col3（NM_000090.3） | F: CCACGGAAACACTGGTGGAC  R: GCCAGCTGCACATCAAGGAC |
| α-SMA（NM_001141945.1） | F：GACAATGGCTCTGGGCTCTGTAA  R：TGTGCTTCGTCACCCACGTA |
| Ki67（NM_032390） | F: ACGCCTGGTTACTATCAAAAGG  R: CAGACCCATTTACTTGTGTTGGA |
| MCM2（NM_004526） | F: ATGGCGGAATCATCGGAATCC  R: GGTGAGGGCATCAGTACGC |
| PCNA（NM_002592） | F: CCTGCTGGGATATTAGCTCCA  R: CAGCGGTAGGTGTCGAAGC |





**Figure S1. Coexpression network of control HSFs (A) and PTX treated HSFs (B).** Genes in red, pink, and yellow are with higher K-core value, while light blue and purple are relative low. The difference of K-core_lA-Bl_ represents the gene regulatory network changes by PTX.
